# New Insights Into Blood-Brain Barrier Maintenance: The Homeostatic Role of β-Amyloid Precursor Protein in Cerebral Vasculature

**DOI:** 10.3389/fphys.2020.01056

**Published:** 2020-08-27

**Authors:** Emma Ristori, Sandra Donnini, Marina Ziche

**Affiliations:** ^1^Department of Life Sciences, University of Siena, Siena, Italy; ^2^Department of Medicine, Surgery and Neuroscience, University of Siena, Siena, Italy

**Keywords:** blood-brain barrier, β-Amyloid precursor protein, Alzheimer’s disease, cerebral amyloid angiopathy, vascular homeostasis, β-Amyloid

## Abstract

Cerebrovascular homeostasis is maintained by the blood-brain barrier (BBB), a highly selective structure that separates the peripheral blood circulation from the brain and protects the central nervous system (CNS). Dysregulation of BBB function is the precursor of several neurodegenerative diseases including Alzheimer’s disease (AD) and cerebral amyloid angiopathy (CAA), both related to β-amyloid (Aβ) accumulation and deposition. The origin of BBB dysfunction before and/or during CAA and AD onset is not known. Several studies raise the possibility that vascular dysfunction could be an early step in these diseases and could even precede significant Aβ deposition. Though accumulation of neuron-derived Aβ peptides is considered the primary influence driving AD and CAA pathogenesis, recent studies highlighted the importance of the physiological role of the β-amyloid precursor protein (APP) in endothelial cell homeostasis, suggesting a potential role of this protein in maintaining vascular stability. In this review, we will discuss the physiological function of APP and its cleavage products in the vascular endothelium. We further suggest how loss of APP homeostatic regulation in the brain vasculature could lead toward pathological outcomes in neurodegenerative disorders.

## Introduction

The brain vasculature is characterized by the presence of the blood-brain barrier (BBB), a specialized structure that maintains the separation between the circulating blood and central nervous system (CNS). The BBB surrounds most of the vessels in the brain and is characterized by the establishment of specialized endothelial tight junctions that consist of transmembrane proteins (claudin3/5, occludins, and JAMs) and cytoplasmic scaffolding proteins (e.g., ZO-1, ZO-2, and ZO-3), involved in cell-to-cell contacts and interacting with actin cytoskeleton and associated proteins (G-proteins, protein kinases, and small GTPases). The tightly interconnected endothelial cells that form the inner lining of BBB vessels not only deliver nutrients and oxygen to brain tissues to ensure neuronal function but also protect the brain by limiting entry of toxins, pathogen, and inflammatory cells. BBB is not the same in all the regions through the brain. While most blood vessels in the brain are situated in the BBB, there are some regions, like the circum-ventricular organs where the microvessels are permeable and lack this specialized structure, allowing the free passage of substances (Wilhelm et al., 2016). The BBB is an essential part of the neurovascular unit (NVU), defined as a complex functional and anatomical structure composed of BBB endothelium, basement membranes (basal lamina and extracellular membranes), astrocytes, pericytes, microglial cells, and neurons. In this context, the neuronal-vascular interaction is critical for proper brain function, and the structural and functional integrity of blood vessels is essential to maintain appropriate brain perfusion and to preserve normal neurological function. Pathological changes in the NVU, including impairment of neurovascular coupling and BBB dysfunction, are present in a variety of neurovascular diseases (Chou et al., 2015; Cai et al., 2017; Iadecola, 2017; Liu et al., 2019; Nizari et al., 2019; Nortley et al., 2019). Increasing evidence supports the hypothesis that vascular dysfunction plays a major role in β-amyloid (Aβ) diseases such as cerebral amyloid angiopathy (CAA) and Alzheimer’s disease (AD) (de la Torre, 2018; Klohs, 2020). While AD is characterized by formation of amyloid plaques in the brain parenchyma, CAA refers to Aβ deposits on the walls of cerebrovasculature. CAA is a common comorbidity of patients with AD. Though accumulation of neuron-derived Aβ in the brain and vessel walls is considered the primary influence driving CAA and AD pathogenesis, clinical trials based on immunotherapy that target and clear Aβ have failed to reverse cognitive loss. The most-cited explanation for the failure of such trials is that the drugs were given too late in the progression of the disease, when the process of Aβ deposition was advanced and at a point of no return in the progression of the disease. This is due in part to an incomplete understanding of the mechanisms that trigger Aβ aggregation and deposition and a lack of notions about the functional role of this peptide and its precursor protein, the amyloid precursor protein (APP) in the cerebrovascular homeostasis. Interestingly, immunotherapy with bapineuzumab, a monoclonal antibody targeting both fibrillar and soluble Aβ, showed severe vascular adverse events like brain vasogenic edema and microhemorrhages, possibly due to a loss in cerebrovascular integrity (Gustafsson et al., 2018). This suggests that physiological levels of Aβ are necessary for normal vascular homeostasis. Furthermore, several studies showed the importance of Aβ and APP for proper physiological function of endothelium, and APP has been hypothesized to play a protective role in vascular dysfunction. In this work, we summarize these findings and highlight the importance of APP and its metabolites on the normal physiology of the vascular system.

## APP Processing in Health and Disease

**Figure 1:**
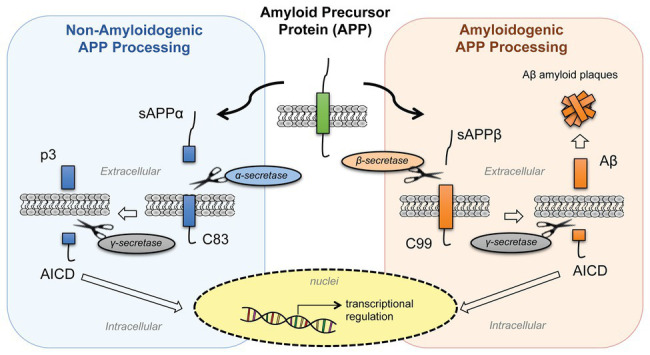
Processing of amyloid precursor protein (APP). APP is processed by two proteolytic pathways: the non-amyloidogenic processing (in blue) and the amyloidogenic processing (in orange). The non-amyloidogenic processing releases in the extracellular space sAPPα and p3 fragments, while the amyloidogenic processing releases sAPPβ and β-amyloid (Aβ) peptides. Both pathways release APP intracellular domain (AICD) fragments into the intracellular space that then translocate to the nuclei and regulate cellular transcription. [sAPPα, soluble amyloid precursor protein; p3, APP non-amyloidogenic extracellular fragment; C83 (βCTF), APP C-terminal fragment beta; AICD, APP intracellular domain; sAPPβ, soluble amyloid precursor protein cleaved by β-secretase; Aβ, beta-amyloid peptide; C89 (αCTF), APP C-terminal fragment alpha].

APP belongs to a conserved gene family that includes two mammalian homologues, the APP-like proteins (APLPs), APLP1 and APLP2. These proteins are type I integral membrane proteins that share similar structural organization and partially overlapping functions, and this may explain why single-gene-knockout animal models have failed to show any major phenotype (Shariati and De Strooper, 2013). Structurally, APP and APLPs share conserved regions, although APP is the only family member containing the sequence encoding Aβ peptides (d’Uscio et al., 2017). APP presents three major isoforms, generated by alternative splicing: APP695, APP751, and APP770. The APP695 isoform is mainly expressed in neurons, whereas APP751 and APP770 are the predominant forms expressed in non-neuronal cells, including endothelial cells and platelets (Van Nostrand et al., 1994). Under physiological conditions, APP is cleaved by different secretases through two main proteolytic pathways: the amyloidogenic and non-amyloidogenic processing (Figure 1). The latter leads to the release in the extracellular space of the soluble form soluble amyloid precursor protein (sAPP-α) generated by α-secretase (ADAM10) cleavage and the p3 peptide through cleavage of α-secretase and the γ-secretase complex (composed of four subunits: presenilins, nicastrin, Aph-1, and Pen-2). By contrast, in the amyloidogenic processing, β-secretase (BACE1) cleavage releases soluble amyloid precursor protein cleaved by β-secretase (sAPP-β; another soluble form with different structure and physiological properties), and subsequent cleavage of BACE1 and γ-secretase generates different Aβ isoforms of various lengths. Moreover, γ-secretase cleavage in the APP transmembrane region yields the biologically active APP intracellular domain (AICD) in both the proteolytic pathways. The main species of Aβ peptides involved in CAA and AD are Aβ1-40 and Aβ1-42. The Aβ1-42 peptides are the predominant form in AD neuronal plaques, whereas deposition of Aβ1-40 peptides on the cerebral vasculature contributes to the onset of CAA (Stakos et al., 2020). While the neuronal origin of Aβ deposits observed in AD and CAA is well established, evidences show that activated endothelial cells and platelets are also able to release Aβ1-40 peptides (Kitazume et al., 2012; Canobbio et al., 2015). Under normal physiological conditions, APP is predominantly processed through the non-amyloidogenic pathway, and the Aβ peptide is constitutively generated at relatively low levels. In addition, several mechanisms of Aβ clearance involving the cerebrovasculature contribute to maintain the concentrations of these peptides to physiological levels in the brain. Some of these mechanisms include Aβ degradation by proteolytic enzymes, phagocytosis by macrophages, intramural periarterial drainage, and receptor mediated Aβ transport across the BBB in which the main transport proteins are: the P-glycoprotein (P-gp), the low-density lipoprotein receptor related protein-1 (LRP-1), and the receptor for advanced glycation end-products (RAGE). While LRP-1 mediates the efflux of Aβ from the brain to the blood circulation, RAGE plays an opposite role by promoting the influx to the brain, thus promoting its accumulation in the parenchyma (Deane et al., 2004; Wan et al., 2014; Wang et al., 2016). Impaired clearance of Aβ across the cerebrovascular endothelium is considered the main cause of CAA (Qi and Ma, 2017). Recent studies suggested the involvement of heparan sulfate proteoglycans (HSPGs) in modulating Aβ clearance and that the altered distribution and increased levels of HSPGs in AD brains might contribute to Aβ aggregation and plaque formation (Zhang et al., 2014; Liu et al., 2016). Interestingly, APP processing is influenced by its cellular distribution: the cell-surface accumulation of APP favors non-amyloidogenic processing (Jiang et al., 2014). On the contrary, the retention of APP in acidic compartments, such as early endosomes, promotes amyloidogenic processing (O’Brien and Wong, 2011). Furthermore, the soluble fragment sAPP-α has a role in the auto regulation of APP processing. One of the proposed mechanisms is that the sAPP-α modulates BACE1 activity promoting the non-amyloidogenic process of APP, thus decreasing Aβ production (Kaden et al., 2008; Gralle et al., 2009; Obregon et al., 2012). All cell types in the NVU express APP and release biologically active APP metabolites, however increasing evidence suggests that dysregulation of APP homeostasis in the brain vasculature would shift the balance toward pathological outcomes. In fact, reduced expression of APP in senescent brain microvascular endothelium contributes to downregulation of sAPPα and promotes the amyloidogenic processing of APP, suggesting that aging-induced loss of APP function might increase the susceptibility to neurovascular dysfunction (Kern et al., 2006; Sun et al., 2018). Moreover, the amyloidogenic secretase BACE1 shows an age-related increased expression and activity in specific brain regions (e.g., cortex) in a mouse model, probably due to alterations of the cellular microenvironment (Chiocco and Lamb, 2007). Not only Aβ production but also its accumulation appears to follow an age-related specific regional pattern (e.g., leptomeningeal and parenchymal vessels), indicating age‐ and spatial-related efficiency of BBB mechanisms for Aβ clearance and degradation (Lewis et al., 2006; van Assema et al., 2012; Zoufal et al., 2020). Based on this evidence, the alteration of APP expression and processing affects the integrity and functionality of neurovascular tissues, and this may be a critical step in the pathogenesis of neurodegenerative diseases.

## APP and Cerebral Vasculature

APP is highly expressed in embryonic endothelium, suggesting an important role of this macromolecule and its metabolites in early angiogenesis (Ott and Bullock, 2001). A strong correlation between APP loss-of-function models and vascular dysfunction has been reported, supporting the importance of this protein and its metabolites on vascular homeostasis. In the zebrafish embryo, knockdown of APP by morpholino causes diffused angiogenic defects especially in the brain. This phenotype can be rescued by reintroducing Aβ peptides supporting the hypothesis that these peptides have an essential role in angiogenesis during embryonic development (Luna et al., 2013). Indeed, blocking Aβ production by inhibition of BACE1 or γ-secretase activity causes reduced angiogenesis both *in vitro* and *in vivo* (Paris et al., 2005). Several studies showed that APP exerts vascular protective properties under physiological conditions. APP regulates expression and function of endothelial nitric oxide synthase (eNOS) in cerebrovascular endothelium (d’Uscio et al., 2018). Loss of APP leads to a loss of eNOS protein expression and to an increase of oxidative stress both *in vivo* and *in vitro* models (d’Uscio et al., 2018; d’Uscio and Katusic, 2019). On the other hand, in eNOS^+/−^ mouse model, reduced availability of endothelial NO leads to increased cerebrovascular concentrations of Aβ (Austin and Katusic, 2020) and transgenic mice overexpressing APP show increased oxidative stress and cerebrovascular dysfunction, associated with altered vasoactive signaling (Tong et al., 2005). In experimental models of stroke, the compensatory increase in cerebrovascular blood flow induced by the occlusion of the common carotid artery is attenuated in APP^−/−^ mice (Koike et al., 2012). Due to this reduced ability to adjust blood flow, APP^−/−^ mice die shortly after the common carotid artery occlusion, whereas wild-type mice survive. Conversely, in transgenic-AD rats, the overexpression of wild-type APP in neuronal tissue exerts neuroprotective effect from ischemic damage (Clarke et al., 2007). APP may also play a role in pathogenesis of atherosclerosis. APP and Aβ can be found in advanced human carotid plaques and in atherosclerotic aortas of Apolipoprotein E-deficient (apoE^−/−^) mice, where the APP overexpression in this mouse model accelerates the development of aortic atherosclerotic (De Meyer et al., 2002; Austin et al., 2009; Tibolla et al., 2010). Moreover, transgenic B6Tg2576 mice overexpressing double Swedish mutated human APP (K670N/M671L) develop non-dietary induced early atherosclerotic (Li et al., 2003). On the contrary, the lack of APP attenuated atherogenesis and leads to plaque stability in double knockout mice APP^−/−^/apoE^−/−^ (Van De Parre et al., 2011). A recent study suggested that the increased pulsatile stretch on the microvessel walls induced by hypertension functions as a mechanic stimulus that modifies the expression and processing of APP, promoting APP overexpression, and favoring APP amyloidogenic processing, and linking APP processing with hypertension (Gangoda et al., 2018). Moreover, modifications of vascular tone could be caused by an alteration of normal neurovascular coupling. Since components of the NVU, such as astrocytes and neurons, control vasodilation by releasing vasoactive molecules, altered vasomotor signals might affect APP processing in cerebral microvasculature (Niwa et al., 2001, 2002; Park et al., 2004, 2005). These studies suggest that several vascular risk factors are linked to APP dysfunction. Although the vascular function of APP has not been defined yet, these evidences suggest the importance of this protein on vascular development and on maintaining the normal tissue homeostasis.

## APP Physiological Roles in Cerebral Vasculature

**Figure 2:**
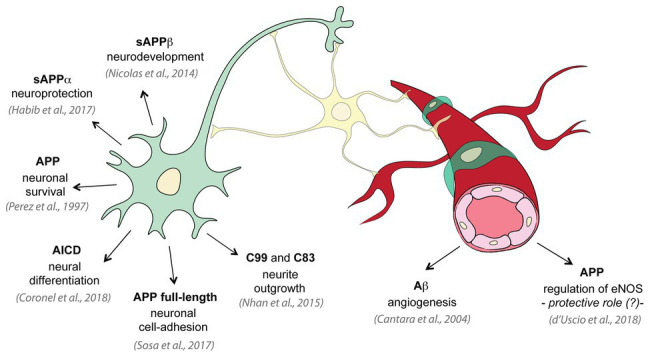
Physiological roles of APP and its metabolites. Several studies investigated the physiological functions of APP on neural tissue: APP promotes neuron viability and axonogenesis (Perez et al., 1997); membrane APP full-length protein shows cell-adhesive properties in neurons (Sosa et al., 2017); sAPPα has been shown to be neuroprotective and to have neuronal trophic properties (Habib et al., 2017); sAPPβ is important for neuronal development (Nicolas and Hassan, 2014); C99 and C83 fragments are involved in neurite outgrowth (Nhan et al., 2015); AICD controls neural differentiation (Coronel et al., 2018). On the contrary only few studies investigated the physiological role of APP in vascular tissue: low concentrations of Aβ promote endothelial cells migration and proliferation and angiogenesis (Cantara et al., 2004); and APP regulates expression of endothelial nitric oxide synthase (eNOS) and may exert a protective role (d’Uscio et al., 2018).

Several physiological roles have been attributed to APP and its processing products, some of which impact neurovascular development and function. Even if APP is notoriously known for its contribution to pathogenesis of neurodegenerative diseases, and many physiological roles have been identified in neural cells (Perez et al., 1997; Nicolas and Hassan, 2014; Nhan et al., 2015; Habib et al., 2017; Coronel et al., 2018), little is known on its function on endothelial cells and cerebral vasculature (Figure 2). Growing evidence suggests that perturbations of some of APP functional activities may contribute to cerebral angiopathy and neurodegeneration (Pearson and Peers, 2006; Thinakaran and Koo, 2008; d’Uscio et al., 2017; Muller et al., 2017). Studying APP cellular functions is complex since both the full-length protein and the secreted or intracellular metabolites have biological activity. The Aβ peptide appears to be involved in protecting the body from various types of infections, sealing leaks in the BBB, improving outcomes after injury, and stimulating angiogenesis by modifying the response to angiogenic factors (Cantara et al., 2004; Brothers et al., 2018). Full-length APP protein has been suggested to function as an important factor for proper migration of neuronal precursors into the cortical plate during the development of mammalian brain (Young-Pearse et al., 2007). The secreted ectodomain sAPP-α is sufficient to rescue prominent deficits in APP^−/−^ mice such as reduction in brain and body weight, impairment in spatial learning, and long-term potentiation (Ring et al., 2007). Moreover, different APP isoforms may have distinct roles in various cell types. While the intracellular C-terminus is conserved in all APP isoforms, the extracellular N-terminus of APP differs between isoforms. The endothelial-specific isoforms, APP751 and APP770, contain two conserved extracellular regions (E1 and E2) connected by an acidic domain (AcD), a Kunitz protease inhibitor (KPI) region, and an OX-2 antigen domain, while the neuronal isoform APP695 lacks the KPI and OX-2 domains. The N-terminal of all APP isoforms presents functional binding sites for metals (zinc and copper) and extracellular matrix (ECM) proteins (heparin, collagen, and laminin; Zheng and Koo, 2011; van der Kant and Goldstein, 2015). Here, we summarize the physiological roles of APP full-length protein and its metabolites in cerebral vasculature.

### APP and Angiogenesis

Many studies have clearly shown the detrimental role of Aβ accumulation on vascular stability; however, evidences suggest that APP mediates endothelial cells’ response to angiogenic growth factors and modulates angiogenesis. Different APP metabolites have been shown to have different roles in angiogenesis and vascular maintenance. Aβ peptides, for example, show both anti-angiogenic and pro-angiogenic effects in a dose-dependent manner *in vitro*. In fact, high micromolar concentrations of different Aβ variants impair angiogenesis, while low nanomolar concentrations of either Aβ1-40 or Aβ1-42 promote angiogenesis in cultured cerebral and peripheral endothelial cells by promoting cell proliferation, migration, and tube formation (Cantara et al., 2004; Cameron et al., 2012). However, the role of Aβ on angiogenesis *in vivo* is still controversial since cerebral hyper-vascularization was observed in human AD brains and transgenic animals overexpressing APP (Biron et al., 2011). To note, the majority of neo-formed vessels observed in AD is so called “string vessels,” non-functional capillaries composed by connecting tissue and lacking endothelial cells (Brown, 2010; Forsberg et al., 2018). It has been proposed that the Aβ-induced aberrant angiogenesis may be the basis for BBB disruption in AD (Biron et al., 2011). Aβ peptides can modulate angiogenesis by functionally interacting with important angiogenic signaling pathways, such as the FGF-2, the vascular endothelial growth factor (VEGF), and the notch signaling (Cantara et al., 2004; Patel et al., 2010; Cameron et al., 2012); however, *in vivo* and *in vitro* studies showed once again controversial results. While brains of patients with AD show upregulation of the VEGF suggesting an interaction of VEGF with APP processing (Burger et al., 2009), *in vitro* studies revealed that high concentrations of Aβ act as a VEGF antagonist, inhibiting VEGR receptor (VEGFR2) activation as well as VEGF-stimulated activation of eNOS in endothelium (Patel et al., 2010; Lamoke et al., 2015; Cho et al., 2017). Another APP metabolite, the secreted APP-α ectodomain, sAPP-α, has been proposed to modulate angiogenesis by binding to the FGF-2 receptor (FGFR-1). In particular, sAPP-α may counterbalance Aβ anti-angiogenic effect by competing with Aβ and FGF-2 for binding to FGFR-1 (Reinhard et al., 2013). More studies are needed to unravel the dichotomy of Aβ roles on angiogenesis and to establish the exact role of each APP metabolite on angiogenesis.

### APP and Cell Adhesion

While the APP soluble metabolites act mostly as ligands, the APP full-length membrane protein functions as a membrane receptor and interacts with cell-adhesion molecules and the ECM. APP extracellular domain binds a series of ECM molecules, including collagen, spondin, laminin, reelin, and HSPGs (glypican and syndecans), and to cell adhesion molecules (CAMs) expressed in neighbor cells, suggesting an important function of APP as an adhesion molecule (Sosa et al., 2017). Many of these interactions stimulate neuronal migration and neurite outgrowth, and we can assume a similar function in endothelial cells (Sosa et al., 2017). The APP extracellular domain is also able to self-dimerize and bind to cell surface receptors, promoting APP surface localization (Deyts et al., 2016). As already discussed, cell-surface accumulation of APP favors non-amyloidogenic processing, thus cell-surface dimerization of APP as well as binding to ECM appears to modulate APP proteolytic processing by secretases. This suggests that APP-dependent adhesive contacts contribute to the control of the dynamics of APP extracellular and intracellular fragments generation. Moreover, APP connects the extracellular environment to the cellular cytoskeleton by physically linking ECM and CAMs elements to the actin cytoskeleton through intracellular scaffold proteins. The cytosolic region of APP presents a YENPTY amino acid sequence motif that is recognized by adaptor proteins (such as Fe65, Mint/X11, and Dab1) capable to link with the actin cytoskeleton and Eb41 (component of cortical cytoskeleton that directly interacts with alpha-actin). Based on this evidence, APP acts as a cell adhesion molecule; however, this physiological role in endothelial cells has not yet been explored.

### APP and Transcription Factors

The AICD plays an important role as a transcriptional regulator and shares many structural and functional similarities with the receptor Notch, a key regulator of endothelial cell phenotype. The cleavage of APP by γ-secretase acts as a receptor processing of APP transmembrane region yielding the biologically active cytosolic fragment AICD that participates in cell signaling. This processing step is shared by many membrane-anchored proteins, included Notch (Kopan and Ilagan, 2004). AICD has a short half-life and is rapidly degraded resulting in low steady-state concentrations. Analogous to Notch receptor signaling, AICD regulates gene expression interacting with transcription factors. In particular, AICD interacts with Fe65 and Tip60 to form a transcriptionally active complex that has been reported to promote glycogen synthase kinase 3 β (GSK3β) gene expression (Cao and Sudhof, 2001; Deyts et al., 2016). APP activates or inhibits GSK3β depending on its subcellular localization: The signaling associated with AICD transcription promotes GSK3β activation, while retaining AICD at the membrane favors inhibition of GSK3β signaling (Chang et al., 2006; Deyts et al., 2012). Dysregulation of GSK3β is involved in several aspects of AD development and progression, pointing out the importance of a correct regulation of APP interacting proteins (Llorens-Martin et al., 2014). Similar to the Notch intracellular domain (NICD), the AICD has been also found to regulate cellular calcium homeostasis through a γ-secretase dependent mechanism (Leissring et al., 2002; Hamid et al., 2007). Notch is an essential protein for endothelial cells, and Notch signaling controls fundamental aspects of angiogenic blood vessel growth and regulates vascular remodeling, vessels stabilization, and endothelial cells quiescence (Mack and Iruela-Arispe, 2018). Similarly, AICD may contribute to the stability of endothelial phenotype by modulating various APP physiological functions including trafficking and signal transduction. The complete understanding of AICD-mediated intracellular molecular mechanisms promoting vascular functionality and BBB integrity requires further investigation.

## Conclusion

APP is a very complex protein that can function as a full-length membrane protein but also through its processing metabolites (included Aβ). While the APP soluble metabolites function mostly as ligands, the APP full-length membrane protein functions as a membrane receptor and interacts with cell-adhesion molecules and the ECM. The processing of this protein through an amyloidogenic or non-amyloidogenic pathway is a key point for the development of amyloid deposits observed in AD and CAA. The failure of clinical trials targeting the amyloidogenic processing and Aβ clearance suggests that more effort is needed to understand the physiological function of APP and modulation of APP processing in cell homeostasis. Even if all cell types of the NVU express APP, experimental *in vivo* and *in vitro* evidence show that endothelial cells dysfunction is related to loss of vascular APP homeostasis. Vascular risk factors such as aging and hypertension can alter APP homeostasis in cerebrovascular tissue not only by modulating APP expression and processing but also by affecting APP protein interaction network. In conclusion, the loss of the physiological activity of APP and its metabolites may have a very important clinical significance. Understanding the influence of APP roles on the functionality of the vascular system might shed light on new therapeutic targets and provide a new perspective on treatment options of neurodegenerative diseases.

## Author Contributions

ER wrote the manuscript. SD and MZ critically reviewed and edited the manuscript. All authors contributed to the article and approved the submitted version.

### Conflict of Interest

The authors declare that the research was conducted in the absence of any commercial or financial relationships that could be construed as a potential conflict of interest.
